# Supplementary material for: Mitochondria dysfunction is one of the causes of diclofenac toxicity in the green alga Chlamydomonas reinhardtii
Source: PeerJ. 2024 Aug 28;12:e18005. doi: 10.7717/peerj.18005 (PMC11365475; doi:10.7717/peerj.18005)

Supplementary Materials to:

Mitochondria dysfunction is one of the causes of diclofenac toxicity in the green alga *Chlamydomonas reinhardtii*

Darya Harshkova[1], Elżbieta Zielińska[1], Magdalena Narajczyk[2], Małgorzata Kapusta[2] and Anna Aksmann[1*]

[1] Department of Plant Experimental Biology and Biotechnology, Faculty of Biology, University of Gdansk, Wita Stwosza 59, 80-308 Gdansk, Poland
[2] Bioimaging Laboratory, Faculty of Biology, University of Gdansk, Wita Stwosza 59, 80-308 Gdansk, Poland

Corresponding Author:
Anna Aksmann[1]
Department of Plant Experimental Biology and Biotechnology, Faculty of Biology, University of Gdansk, Wita Stwosza 59, 80-308 Gdansk, Poland
Email address: anna.aksmann@ug.edu.pl

Table S1:

Mitochondrial membrane potential of *Chlamydomonas reinhardtii*.

DCF was applied to the culture at the beginning of the cell cycle (0h) at a concentration of 135.5 mg·L^{-1}. KCN = potassium cyanide-incubated cells; SHAM = salicylhydroxamic acid-incubated cells; DCF = diclofenac-treated cells. Data shown as mean % of control were originally expressed in arbitrary units (a.u) of MMP. * = statistically significant differences between control and treated populations, $p < 0.05$ (Mann–Whitney U test; n=8).

	0h	SD	3h	SD	6h	SD	9h	SD
**control**	100.0	11.6	100.0	31.4	100.0	50.4	100.0	32.4
**control + KCN**	90.7	20.2	79.2	18.2	75.2	60.8	96.5	17.9
**control + SHAM**	106.3	33.6	71.9	37.3	86.0	24.5	**55.1***	24.4
**control + KCN + SHAM**	96.6	43.8	94.5	23.8	95.0	41.3	**63.4***	7.6
**DCF**	100.0	11.6	77.9	32.3	**45.1***	28.0	**33.3***	17.7
**DCF+ KCN**	90.7	20.2	64.1	22.8	47.8	30.7	40.2	18.5
**DCF + SHAM**	106.3	33.6	79.5	18.3	53.2	30.7	46.4	18.5
**DCF + KCN + SHAM**	96.6	43.8	102.2	16.0	46.6	18.8	45.6	47.5

Table S2:

Correlation matrices (Spearman's test) for values of oxygen consumption rates.

KCN = potassium cyanide-incubated cells; SHAM = salicylhydroxamic acid-incubated cells; DCF = DCF-treated cells. * indicates statistically significant correlation between groups at $p < 0.05$.

	**KCN**	**SHAM**	**KCN + SHAM**	**DCF**
**KCN**	1.000000	-0.085714	-0.600000	0.771429
**SHAM**	-0.085714	1.000000	0.485714	-0.200000
**KCN + SHAM**	-0.600000	0.485714	1.000000	-0.542857
**DCF**	0.771429	-0.200000	-0.542857	1.000000

Table S3:

Discriminant analysis of MMP, oxygen consumption rates, and cell volume in *Chlamydomonas reinhardtii* control cells.

DCF-treated cells and/or with ETC-inhibitors after 6 h from the beginning of the cell cycle. * indicates statistically significant differences between cultures at $p < 0.05$ (Mann–Whitney U test; n = 8).

	Wilks' lambda	partial lambda	F-remove (4.23)	p
MMP, a.u.	**0.3073***	**0.32582***	**11.8997***	$p < 0.05$
oxygen consumption rates, nmol $O_2 \times 10^6$ cells $^{-1} \times$ min $^{-1}$	**0.1696***	**0.5904***	**3.9892***	$p < 0.05$
cell volume, fL	0.1440	0.6954	2.5190	0.0690

Table S4:

Standardization coefficients for canonical variables in the discriminant analysis of MMP, oxygen consumption rates, and cell volume in *Chlamydomonas reinhardtii* control cells.

Cells treated with diclofenac and/or with ETC inhibitors at 6th h of the cell cycle, n = 8.

	**Root 1**	**Root 2**	**Root 3**
**MMP, a.u.**	0.7998	-0.6402	-0.0574
**oxygen consumption rates, nmol $O_2 \times 10^6$ cells $^{-1} \times$ min $^{-1}$**	-0.3580	-0.7610	0.5655
**cell volume,** fL	0.5245	0.3282	0.8017

Table S5:
Correlation matrices (Spearman's test) for values of MMP.

KCN = potassium cyanide-incubated cells; SHAM = salicylhydroxamic acid-incubated cells; DCF = DCF-treated cells. * indicates statistically significant correlation between groups at p < 0.05.

	KCN	SHAM	KCN + SHAM	DCF
**KCN**	1.0000	0.4196	0.1468	0.3636
**SHAM**	0.4196	1.0000	0.0909	-0.0559
**KCN + SHAM**	0.1468	0.0909	1.0000	**0.65035***
**DCF**	0.3636	-0.0559	**0.65035***	1.0000

Figure S1:
Mean fluorescence intensity measurements in JC-1 staining *Chlamydomonas reinhardtii* cells (confocal microscopy).
DCF was applied to the culture at 0 h at a concentration of 135.5 mg × L^{-1}. C-red cells = control material, visible chloroplast and red fluorescent J-aggregates (peak emission ~570 nm); DCF = cells treated with DCF for 24h, some cells show mainly red fluorescence of J-aggregates like control material (DCF- red cells), some show green fluorescence emission at (~517 nm) for the monomeric form of the cells (DCF-green cells). Data are presented as mean ± SD. * indicates statistically significant differences between control and treated populations (p < 0.05; Mann–Whitney U test; n = 10, each cell was measured in triplicate). Photo credit: Małgorzata Kapusta.

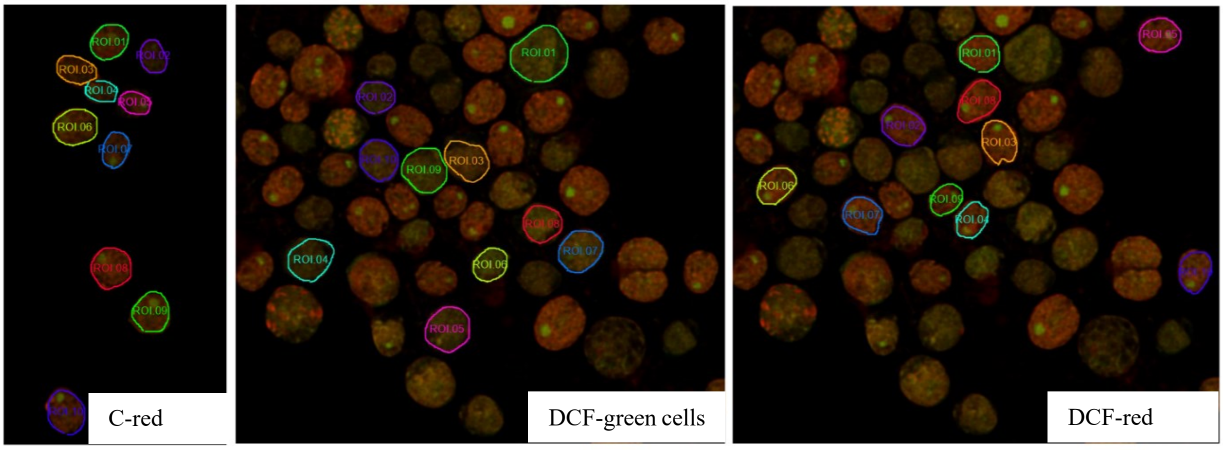

Monomers (FITC, Ex 495/Em 517 nm)			
	**C-red cells**	**DCF-green cells**	**DCF-red cells**
**mean ±SD**	554.48 ±158.54	490.78 ± 133.06	494.94 ± 116.42
**Oligomers (TRITC, Ex 532/ Em 570 nm)**			
	**C-red cells**	**DCF-green cells**	**DCF-red cells**
**mean ±SD**	3124.21 ± 745.92	**2243.17 ± 626.86 ***	3427.38 ± 607.91

Figure S2:

Visualization of JC-1 fluorescence and autofluorescence of chlorophyll in *Chlamydomonas reinhardtii* cells, control (A) and treated with DCF (B) (confocal microscopy).

DCF was applied to the culture at 0 h at a concentration of 135.5 mg × L^{-1}. Multicolor imaging of control (A) and with 24h DCF-treatment (B) of *Chlamydomonas reindhardti*. Green and red fluorescence represent monomers (max. emission ~529 nm) and oligomers (aggregates) (max. emission ~590 nm) visualized using JC-1, respectively. Merged photos represent JC-1 staining combined with autofluorescence of chlorophyll (blue fluorescence). Photo credit: Małgorzata Kapusta.

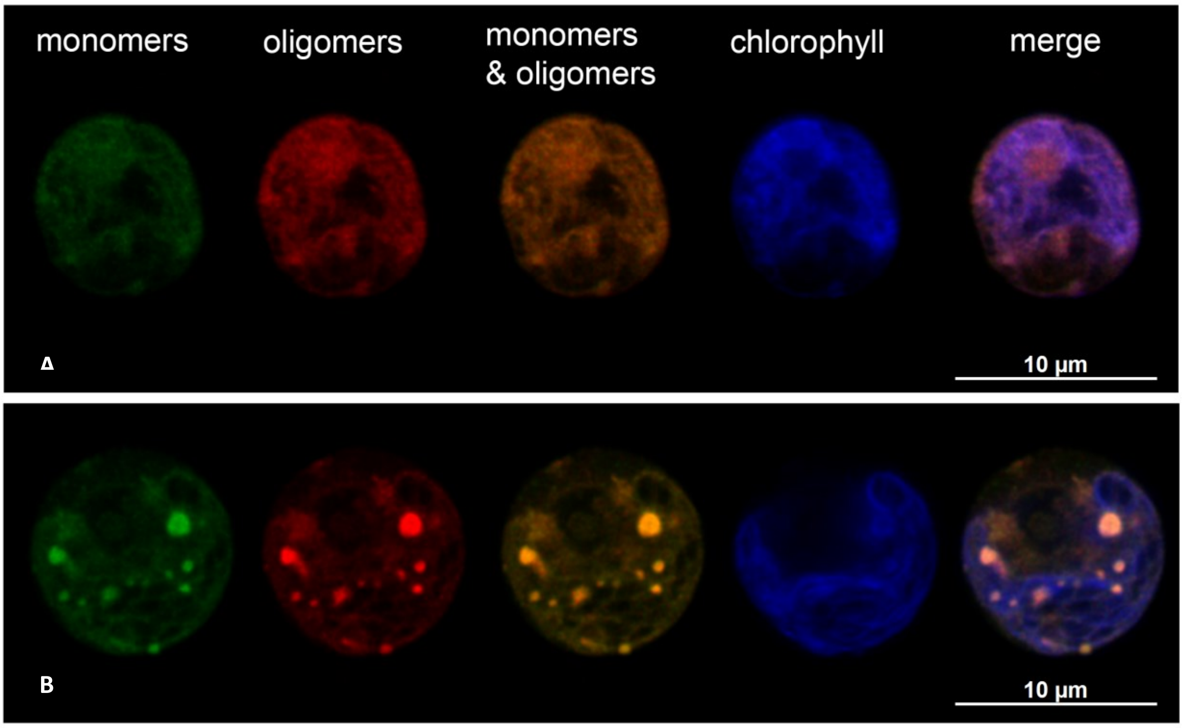

Figure S3:

Visualization of MitoTracker™ Orange CM-H2TMRos fluorescence and autofluorescence of chlorophyll in *Chlamydomonas reinhardtii* cells (confocal microscopy).

Mitochondria of *Chlamydomonas reindhardti* stained with MitoTracker™ Orange CM-H2TMRos (yellow fluorescence). Merged photo represent mitochondria staining combined with autofluorescence of chlorophyll (blue fluorescence). Photo credit: Małgorzata Kapusta.

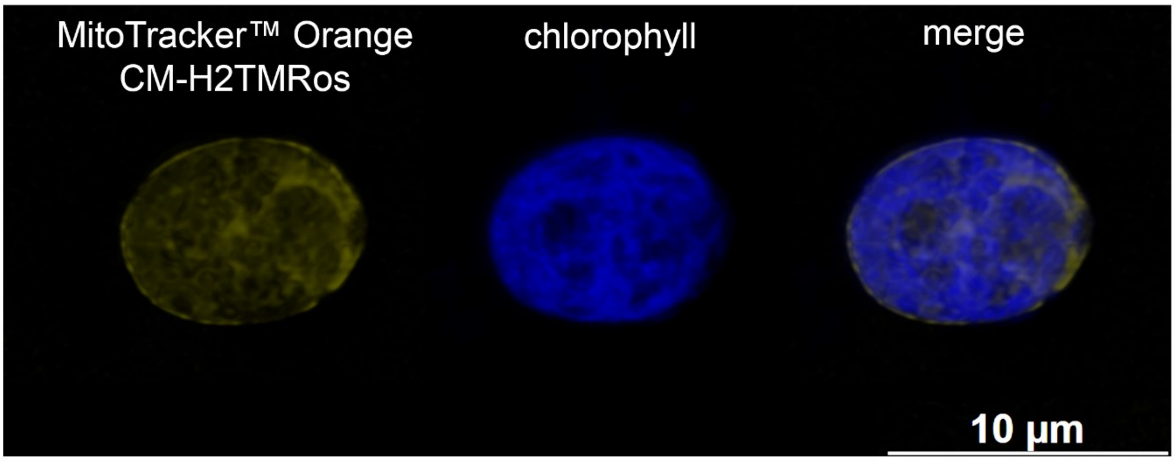

Supplement: Supplemental Information 2 — Table S1: Mitochondrial membrane potential of Chlamydomonas reinhardtii. Table S2: Correlation matrices (Spearman’s test) for values of oxygen consumption rates. Table S3: Discriminant analysis of MMP, oxygen consumption rates, and cell volume in Chlamydomonas reinhardtii control cells. Table S4: Standardization coefficients for canonical variables in the discriminant analysis of MMP, oxygen consumption rates, and cell volume in Chlamydomonas reinhardtii control cells. Table S5: Correlation matrices (Spearman’s test) for values of MMP. Figure S1: Mean fluorescence intensity measurements in JC-1 staining Chlamydomonas reinhardtii cells (confocal microscopy). Figure S2: Visualization of JC-1 fluorescence and autofluorescence of chlorophyll in Chlamydomonas reinhardtii cells, control (A) and treated with DCF (B) (confocal microscopy). Figure S3: Visualization of MitoTracker™ Orange CM-H2TMRos fluorescence and autofluorescence of chlorophyll in Chlamydomonas reinhardtii cells (confocal microscopy). [file peerj-12-18005-s002.pdf]
