# Supplementary material for: Mitochondria dysfunction is one of the causes of diclofenac toxicity in the green alga Chlamydomonas reinhardtii
Source: PeerJ. 2024 Aug 28;12:e18005. doi: 10.7717/peerj.18005 (PMC11365475; doi:10.7717/peerj.18005)
Supplement: Supplemental Information 3 [file peerj-12-18005-s003.pdf]

Dear Editors,

in the revised version of the manuscript "Mitochondria dysfunction is one of the causes of diclofenac toxicity in the green alga *Chlamydomonas reinhardtii*", dr Małgorzata Kapusta has been added as an co-author. Her co-authorship is a result of suggestions of Reviewer#2, who asked for additional, new experiments to visualize cells with confocal microscopy (Q2 in Reviewer#2 comments). Since none of the authors included in the original version is an expert in confocal microscopy, we invited Dr. Kapusta, who is an expert in this field, to cooperate with us.

In the revised version of the manuscript all parts related to confocal microscopy are a result of dr Kapusta work, i.e., confocal microscopy analysis shown in photographs and fluorescence signal analyses (Figure 4, Figure S1 in the revised version) along with their description and interpretation (in Results and Discussion) and methods description (second paragraph in subchapter "Mitochondrial membrane potential (MMP) and mitochondrial ROS (mtROS) assessment"). All parts of manuscript that are related to dr Kapusta's contribution have been marked in the manuscript, in the version with changes tracked.

I would like to mention, that all co-authors approved dr Kapusta as co-author, which has been confirmed using change confirmation emails.

With best regards,
Anna Aksmann
